# Association between neurofilament light chain concentrations and outcomes in patients with moderate to severe traumatic brain injury: a systematic review and meta-analysis

**DOI:** 10.1186/s13054-026-06036-3

**Published:** 2026-05-04

**Authors:** Marwan Bouras, Mathieu Pageau, Marc-Aurèle Gagnon, Olivier Costerousse, Karolanne Demers, Anouk Grenier-Gagnon, Chartelin Jean Isaac, Tomas Hayg Torkomyan, François Lauzier, Ryan Zarychanski, Charles L. Francoeur, Peter Gerges, Godwill Abiala, Lynne Moore, Shane W. English, Alexis F. Turgeon

**Affiliations:** 1 CHU de Québec, Population Health and Optimal Health Practices Research Unit Trauma-Emergency-Critical Care Medicine, Université Laval Research Center, Université Laval, Québec City, QC Canada; 2 Department of Anesthesiology and Critical Care Medicine, Division of Critical Care Medicine, Université Laval, Québec City, QC Canada; 3 Department of Anesthesiology and Critical Care Medicine, Centre Hospitalier Régional Universitaire de Brest, Brest, France; 4 Department of Medicine, Université Laval, Québec City, QC Canada; 5 Department of Internal Medicine, Sections of Critical Care Medicine, of Hematology and of Medical Oncology, Rady Faculty of Medicine, University of Manitoba, Winnipeg, MB Canada; 6 Research Institute of Oncology and Hematology, CancerCare Manitoba, Winnipeg, MB Canada; 7 Department of Preventive and Social Medicine, Université Laval, Québec City, QC Canada; 8 Acute Care Research, Ottawa Hospital Research Institute, Ottawa, ON Canada; 9 Division of Critical Care, Department of Medicine, University of Ottawa, Ottawa, ON Canada

**Keywords:** Neurofilament light chain, Traumatic brain injury, Neurological outcomes, Mortality, Glasgow Outcome Scale.

## Abstract

**Background:**

Moderate to severe traumatic brain injury (TBI) is associated with high rates of mortality and long-term disability. Accurate biomarkers are needed to predict longterm neurological outcomes and guide decision-making early after TBI. Neurofilament light chain (NfL), a structural protein of neurons, has emerged as a promising candidate, but its association with outcomes in this population remains uncertain.

**Methods:**

We conducted a systematic review and meta-analysis to assess the association between blood or cerebrospinal fluid NfL concentrations and outcomes in adults with moderate to severe TBI. We searched MEDLINE, Embase, Cochrane CENTRAL and Web of Science from inception to October 2025. Eligible studies included cohort studies or randomized controlled trials reporting NfL levels measured during the acute phase and reporting at least one outcome of interest. Our primary outcome was long-term neurological function, defined as the latest available Glasgow Outcome Scale (GOS) or Glasgow Outcome Scale–Extended (GOS-E) score, dichotomized into unfavorable (GOS ≤ 3 or GOS-E ≤ 4) and favorable (GOS > 3 or GOS-E > 4). Mortality, at any time point, was a secondary outcome. Risk of bias was assessed using an adapted scale from the QUADAS-2 tool, and certainty of evidence was evaluated using GRADE criteria.

**Results:**

Fourteen studies (2,905 participants) were included, with ten (*n* = 1,648) contributing to the meta-analysis for our primary outcome. Higher NfL concentrations were associated with unfavorable neurological outcomes, with moderately higher levels in patients with poor outcomes compared with those with favorable outcomes (SMD 0.45, 95% CI 0.33–0.56; I² = 12%). Six studies (*n* = 483) assessed mortality; higher NfL concentrations were associated with increased mortality (SMD 0.71, 95% CI 0.04–1.39; I² = 82%), with a more consistent association when NfL was measured within 24 h after injury (I² = 0%). The certainty of evidence was graded as very low for both outcomes, reflecting risk of bias and, for mortality, additional inconsistency and imprecision.

**Conclusions:**

Higher NfL concentrations were associated with unfavorable neurological outcomes after moderate-to-severe TBI. The association with mortality was more uncertain and should be interpreted with caution given the substantial heterogeneity across studies. Its incremental prognostic value beyond known predictors remains uncertain.

**Trial registration:**

PROSPERO CRD42022332110, 22 May 2022.

**Supplementary Information:**

The online version contains supplementary material available at 10.1186/s13054-026-06036-3.

## Background

Moderate to severe traumatic brain injury (TBI) is frequently associated with high mortality and long-term disability [1]. Early after injury, patients are unconscious or unable to express their wishes, and families and clinicians must make critical decisions about continuing life-sustaining treatments [2]. In this context, accurate biomarkers have been proposed to help predict outcomes and support decision-making [3]. Biomarker research in TBI has shown that several markers are associated with clinical outcomes. Previous systematic reviews have focused on the prognostic value of neuronal markers such as neuron-specific enolase (NSE) [4] and S100 calcium-binding protein B (S100B) [5], and glial markers such as glial fibrillary acidic protein (GFAP) [6]. Even if an association between blood levels of these biomarkers and neurological recovery was observed, the lack of standardized thresholds and methodological variability limit their clinical applicability. Therefore, in parallel with efforts to validate and standardize existing biomarkers, expanding the evaluation to additional cerebral biomarkers may help improve outcome prediction in this population.

Neurofilament light chain (NfL) is a cytoskeletal protein located in the cytoplasm of neurons, where it plays a key role in the growth, structural maintenance, and stability of large-caliber myelinated axons [7]. Its elevation has been consistently reported across a wide spectrum of neurological disorders, [8] supporting its value as a general diagnostic biomarker for neurodegenerative diseases [9, 10]. In the context of moderate or severe TBI, serum concentrations of NfL may reflect its release from damaged white matter. NfL has been shown to correlate with ventricular cerebrospinal fluid (CSF) levels [11] and remain detectable in the blood longer than other biomarkers such as S100B or NSE [12]. NfL levels reflect the extent of brain damage, as confirmed by advanced MRI, cerebral microdialysis, and experimental models [11]. Previous systematic reviews and meta-analyses investigating NfL in patients with TBI have focused on its diagnostic value in mild TBI, showing higher levels compared to healthy individuals [13, 14]. However, whether NfL concentrations are associated with long-term neurological outcomes and mortality in patients with moderate to severe TBI remains unclear. To address this gap, we conducted a systematic review and meta-analysis to evaluate whether NfL concentrations are associated with long-term functional outcomes and mortality in adults with moderate to severe TBI.

## Methods

### Study design

Our systematic review and meta-analysis was conducted following the guidance provided by the Cochrane Collaboration recommendations [15]. The protocol was registered on PROSPERO (CRD42022332110). Our results are reported according to the Preferred Reporting Items for Systematic Reviews and Meta-Analyses (PRISMA) statement [16].

### Eligibility criteria

We included retrospective or prospective cohort studies and randomized controlled trials reporting on NfL concentrations measured in adult patients (≥ 18 years) with acute moderate (Glasgow Coma Scale (GCS) score 9 to 12) or severe (GCS score 3 to 8) TBI. Studies were eligible if they reported at least one of our outcomes of interest. We excluded studies in which more than 50% of patients had mild TBI (GCS ≥ 13), unless individual patient data allowed stratified analysis. All types of NfL sampling and measurement (blood or CSF) were considered; no language restrictions were applied. In cases where multiple articles reported on the same population, we included the study with the largest sample size.

### Outcomes

Our primary outcome was long-term neurological function, defined as the latest available Glasgow Outcome Scale (GOS) [17] or Glasgow Outcome Scale–Extended (GOS-E) score [18], dichotomized as unfavorable (GOS ≤ 3 or GOS-E ≤ 4) and favorable outcome (GOS > 3 or GOS-E > 4). Secondary outcomes included mortality – at any time point, length of stay in hospital and intensive care unit, and quality of life.

### Search strategy

We searched MEDLINE (via Ovid), Embase (via Ovid), Cochrane Central Register of Controlled Trials (CENTRAL), and Web of Science from inception to October 24, 2025. Our search strategy was developed with an information specialist using broad text and index terms to optimize sensitivity and specificity for prognostic studies [19, 20], and validated according to the Peer Review of Electronic Search Strategies (PRESS) 2015 guidelines [21]. The full search strategy for MEDLINE is available in Appendix 1. We also screened abstracts from relevant meetings, reference lists of included studies, and prior reviews.

### Study selection and data extraction

We managed citations and duplicates using Covidence systematic review software (Cochrane, Melbourne, 2020 version). Pairs of reviewers (M-A.G., C.J.I., T.H.K., K.D., A.G-G.) independently screened studies and extracted data, with disagreements resolved by a third reviewer (AFT). We collected trial characteristics (e.g., country, study design, sample size), patient data (e.g., age, sex, GCS on admission), presence or absence of extracerebral injuries, NfL measurement (e.g., assay type, sample type, timing), and outcome information (e.g., outcome definitions, time points).

### Risk of bias assessment

We assessed the methodological quality of included studies using a quality assessment scale adapted from the Quality Assessment of Diagnostic Accuracy Studies tool (QUADAS-2) [22] for prognostic studies. This modified tool included assessment of patient selection, NfL measurement, outcome assessment, flow and timing, confounding, and applicability concerns, and has been previously applied in published prognostic biomarker systematic reviews [4–6]. We performed a separate assessment of reporting quality using the STROBE recommendations [23]. Risk of bias and reporting quality were evaluated independently by two reviewers (M-A.G., C.J.I., K.D., A.G-G., M.P.) with adjudication by a third reviewer in case of disagreement (AFT).

### Statistical analysis plan

All analyses were conducted using Review Manager version 5.4.1 (RevMan, The Cochrane Collaboration, Oxford, United Kingdom). We used the Der Simonian and Laird random-effects models and the inverse variance method to assess associations between continuous NfL concentrations and dichotomized outcomes (neurological outcome and mortality). Expecting measurement using different biological matrices, assay methods, and concentration ranges,* w*e reported treatment effects as standardized mean differences (SMD) with 95% confidence intervals (CI). The SMD expresses the difference in means between groups divided by the pooled standard deviation, allowing comparison across studies that measured the same outcome on different scales. A positive SMD indicates higher NfL concentrations in patients with unfavorable outcomes compared with those with favorable outcomes. An SMD of 0.2 is generally considered a small effect, 0.5 a moderate effect, and 0.8 a large effect [24, 25]. Presence of statistical heterogeneity was assessed using the I² statistic and interpreted according to the Cochrane Handbook with I^2^ > 50% representing high heterogeneity [26]. Pre-specified subgroup analyses explored potential heterogeneity by timing of NfL sampling (initial 24 h vs. any time), type of sample (blood vs. CSF), TBI severity (moderate & severe vs. severe TBI), and assay technique (SIMOA vs. ELISA). For all statistical tests, we applied a two-tailed α level of 0.05.

### Strength of evidence

Two reviewers independently assessed the certainty of the evidence for each outcome (M-A.G., C.J.I.), in accordance with the GRADE (Grading of Recommendations Assessment, Development and Evaluation) Working Group guidance [27]. Assessments were performed using the GRADEpro Guideline Development Tool (GRADEpro GDT, McMaster University, Ontario, Canada).

## Results

**Fig. 1:**
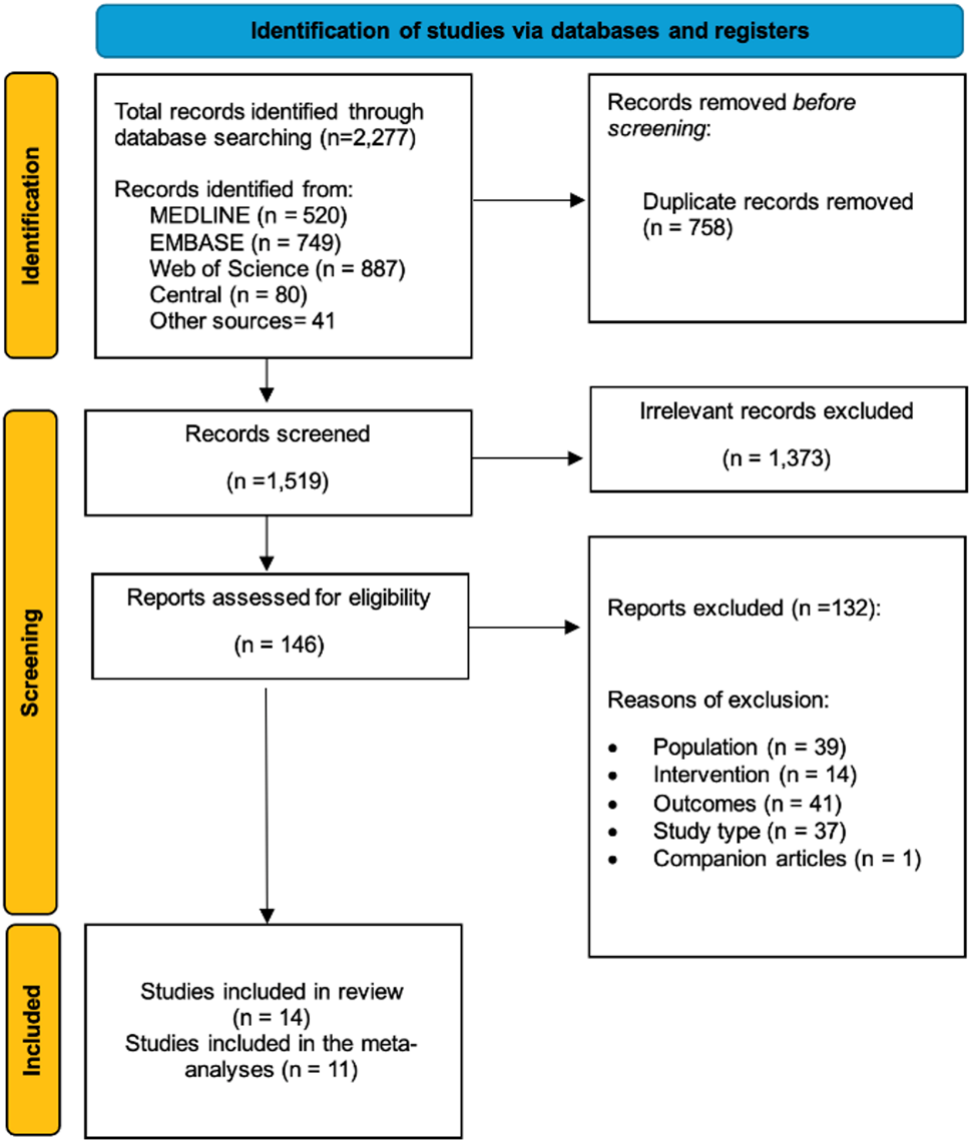
Flow diagram of included studies

Our search strategy retrieved 1,876 citations. After removing duplicates, 1,259 citations remained and were screened; 113 full-text articles were evaluated (Fig. 1). One publication [28] shared the same patient cohort with an included study [29] and was thus considered a companion article. Fourteen studies (2,905 participants) published between 2015 and 2025 were included [11, 29–40]. All included studies were peer-reviewed except for one conference abstract [34].

### Characteristics of studies

All 14 included studies were observational cohort studies [11, 29–40]. Nine studies were single-center, five conducted in Sweden [30–33, 35, 37] one in Cameroon [36], one in India [41] and two in Finland [38, 39] and three were multicenter studies [11, 29, 40]. Seven studies reported only serum concentrations of NfL [29–32, 36, 37, 41], 4 studies reported plasma concentrations [11, 34, 38, 39], one reported only CSF measurements [35] and 2 studies reported both CSF and serum concentrations [33, 40]. Initial NfL sampling occurred between 14 h and 11 days post-injury, with final measurements taken between 24 h and 15 days post-injury. All studies defined unfavorable outcomes using the GOS or the GOS-E (Table 1). Outcome assessments occurred between 6- and 12-months post- injury. Study characteristics are presented in Table 1.

**Table 1:** Characteristics of included studies

Studies	Study characteristics			Patient characteristics						NfL sampling			Timing of outcome assessment *	
	**Country**	**Cohort study**	**Number of centers**	**Patients** n	**Sex** M: F	**Age** Mean (SD) *Median [IQR]*	**GCS on admission** Mean (SD) *Median [IQR]*	**TBI severity**		**Sampling time**	**Sample type**	**Biochem. assay**	**Primary outcome (GOS/GOS-E)**	**Mortality**
								**Severe** n (%)	**Moderate** n (%)					
Studies included in meta-analyses (*n* = 11)														
Ljungqvist et al. 2017 [31]	Sweden	Prosp.	Single	8**	7:1	43.13 (16.75) 43.5 *[28–55.5.5]*	5.4 (2.3) *5.5 [3.5–6.5]*	7 (87.5%)	1 (12.5%)	4–9 days post-injury	Serum	SIMOA	GOS-E 12 mo	12 mo
Al Nimer et al. 2015 [33]	Sweden	Retro.	Single	158**	NR	49 (17.6) *53 [35–63]*	5.9 (2.9) *6[3–7]*	128 (81%)	30 (19%)	1–15 days PT	Serum, CSF	ELISA	GOS 6–12 mo	6–12 mo
Shahim et al. 2016 [30]	Sweden	Prosp.	Single	70	NR	- *36 [22–54]*	- *[Min-Max: 3–8]*	70 (100%)	0	0–12 days post-trauma	Serum	Ultrasensitive ELISA (using SIMOA platform)	GOS 12 mo	12 mo
Andersson et al. 2024 [35]	Sweden	Retro.	Single	47	4:1	45.4 (16.1)	-	47 (100%)	0	0–18 days	CSF	ELISA	GOS-E 12 mo	12 mo
Buh et al. 2023 [36]	Cameroon	Prosp.	Single	160	9:1	*32 [26–39]*	*12 [8–14]*	39 (24%)	55 (34%)	24 h post trauma	Serum	ELISA	GOS-E 6 mo	6 mo
Gonzalez-Ortiz et al. 2023 [37]	Sweden	Prosp.	Single	39	3:1	*47 [29–64]*	*[Min-Max: 3–8]*	39 (100%)	0	0–7 days post-trauma	Serum	SIMOA	GOS 12 mo	NR
Korhonen et al. 2023 [38]	Finland	Prosp.	Single	80	3:1	54.9 (19.4)	*11 [min-max:3–15]*	37 (43.5.3%)	48 (56.5%)	24 h post admission	Plasma	SIMOA	GOS-E 6–12 mo	NR
Richter et al. 2023 [29]	Multiple countries	-	Multicenter	872	1:1	47 (29–64)	NR	644 (74%)	228 (26%)	14 h PT	Serum	SIMOA	GOS-E 6 mo	NR
Tuure et al. 2023 [39]	Finland	Prosp.	Single	67	3:1	52.1 (19.2)	NR	26 (39%)	39 (28%)	9 mo	Plasma	SIMOA	GOS-E 9 mo	NR
Wang et al. 2024 [40]	USA	Prosp.	Multicenter	382	3:1	*36.35 (20.2)*	6.8 (1.8)	224 (58.6%)	-	0–6 days post injury	Serum, CSF	SIMOA	GOS-E 6 mo	NR
Pasupuleti et al. 2025 [41]	India	Prosp.	Single	62	59:3	3–8: 32.1 (10.8) 9–13: 39.53 (18.7)	NR	34 (54.8)	28 (45.2)	24–36 h	Serum	ELISA	NR	Unclear
Studies not included in meta-analyses (*n* = 3)														
Thelin et al. 2019 [32]	Sweden	Prosp.	Single	172	130: 42	*-* *55 [38–62]*	NR	121 (70%)	GCS 9–13: 38 (22%)	Median 3-, 6- and 9-days PT	Serum	N4PA. SIMOA	GOS 12 mo	NR
Graham et al. 2021 [11]	Multiple countries	Prosp.	Multicenter	197	152:45	*47 [30]*	3–8: *N* = 89 (45.2) 9–13: *N* = 55 (27.9) 14–15: *N* = 51 (25.9)	89 (45.2%)	GCS 9–13: 55 (27.9%)	NR	Plasma	SIMOA	GOS-E 6–12 mo	NR
Lynch et al. 2021 [34]	USA	Prosp	NR	591	NR	*NR*	NR	NR	NR	Within 24 h	Plasma	NR	GOS-E	NR

* Timing of assessment of the dichotomous outcome (functional outcome or mortality) in association with reported mean (SD) or median (IQR) measured level of NFL (in the ER or the ICU)

** Individual patient data were available, so only moderate and severe TBI patients were included in the meta-analyses

Biochem. assay: Biochemical assay; CSF: Cerebrospinal fluid; GCS: Glasgow Coma Scale; GOS: Glasgow Outcome Scale; GOS-E: Glasgow Outcome Scale – Extended

H. Hours; Multi : Multicenter; Mo: Months; N/A: not applicable (the study is not included in the meta-analyses); NR: Not reported; Prosp.: Prospective; PT: Post-trauma; Retro.: Retrospective

mo: month; Simoa Human Neurology 4-Plex A, ELISA: Enzyme-Linked Immunosorbent Assay

### Risk of bias

Three studies were judged at high risk of bias because of unclear consecutive enrollment procedures [31, 33, 38] and one study was judged at high risk of confounding bias because it did not adequately account for important factors, particularly age (Figure S1) [31]. One study was at high risk of bias because not all enrolled patients were included in analyses [30]. All studies had low applicability concerns except the conference abstract, which provided insufficient information [34]. Reporting quality, based on STROBE, varied across studies (Table S1). Basic items (objectives, participants, and main results) were consistently reported in five studies [11, 29, 30, 39, 42]. In contrast, several items were incompletely addressed. Sample size calculation was only reported in two studies [11, 42]. Reporting of strategies to address bias was limited, with sufficient details provided in eight studies [28, 29, 32, 33, 35, 36, 39, 42]. Information on missing data and loss to follow-up was explicitly presented in four studies [30–33]. Flow diagrams were rarely included [11, 30]. We found no evidence of publication bias for the primary outcome based on visual inspection of the funnel plot (Figure S2).

### Primary outcome

**Fig. 2:**
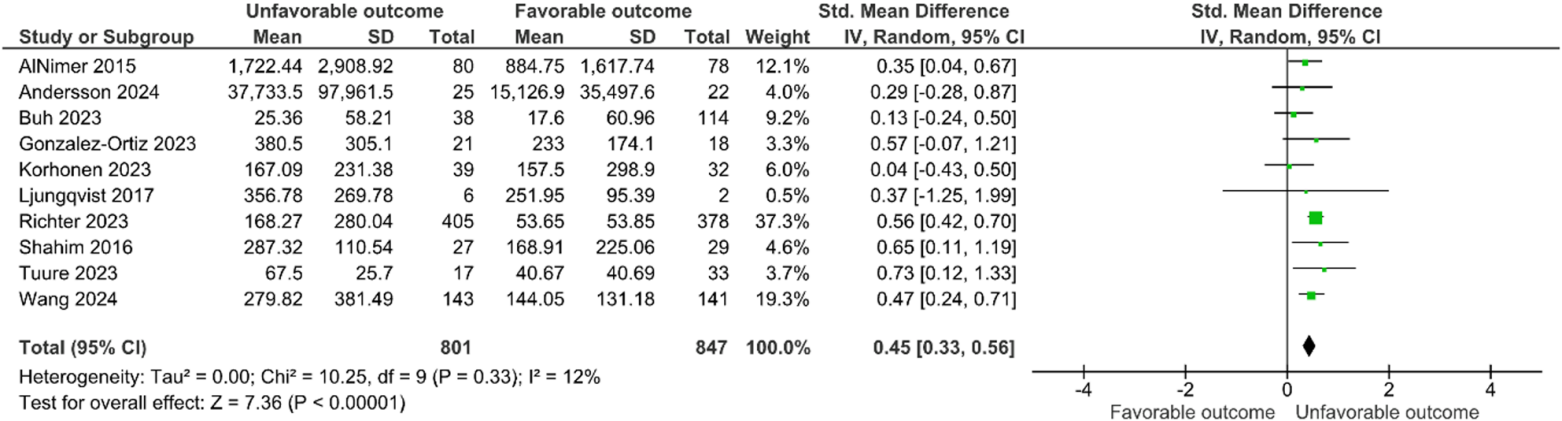
Association between NfL levels and unfavorable neurological outcome. Legend: CI = confidence interval; SD = standard deviation; IV = inverse variance

Ten studies (*n* = 1,648 participants) [29–31, 33, 35–40] were included in the meta-analysis evaluating the association between NfL concentrations and unfavorable neurological outcomes. Elevated NfL concentrations were significantly associated with unfavorable outcomes (standardized mean difference (SMD) 0.45 [95% CI 0.33 to 0.56]; I² = 12%) (Fig. 2). Subgroup analyses failed to identify convincing evidence of heterogeneity according to sampling time, TBI severity, type of sample, and type of assay (Table 2, Figure S3 A to D). Three studies could not be included in the meta-analysis; two concluded a correlation between NfL and functional outcomes without providing effect sizes or statistical significance [34, 35], while one indicated that NfL enhanced the predictive ability of IMPACT models alongside other biomarkers [32]. As all included studies were observational, certainty of evidence started at a low level and was further downgraded for risk of bias, mainly because of concerns regarding patient selection and uncertainty in some outcome and confounding assessments, resulting in an overall very low certainty rating (Table 3).

**Table 2:** Subgroup analyses for unfavourable neurological outcome and mortality

	No of studies	No. of patients	Standardized Mean difference (95%CI)	I^2^
**Unfavorable neurological outcome**				
*Overall*	10	1648	0.45 (0.33 to 0.56)	12%
*Subgroups*				
Sampling time				
Initial 24 h	3	991	0.45 (0.15 to 0.74)	59%
All time	7	657	0.40 (0.25 to 0.56)	0%
TBI severity				
Moderate and severe	7	1506	0.41 (0.25 to 0.57)	36%
Severe	3	142	0.51 (0.17 to 0.84)	0%
Type of sample				
Blood	9	1601	0.44 (0.31 to 0.57)	19%
CSF	1	47	0.29 (−0.28 to 0.87)	NA
Type of biochemical assay				
SIMOA	6	1235	0.51 (0.40 to 0.63)	1%
ELISA	4	413	0.32 (0.11 to 0.52)	0%
**Mortality**				
*Overall*	6	483	0.71 (0.04 to 1.39)	82%
*Subgroups*				
Sampling time				
Initial 24 h	2	208	0.58 (0.18 to 0.98)	0%
All time	4	275	0.78 (−0.43 to 1.99)	89%
TBI severity				
Moderate and severe	4	380	0.60 (−0.30 to 1.50)	86%
Severe	2	103	0.91 (−0.39 to 2.22)	81%
Type of sample				
Blood	5	436	0.54 (−0.18 to 1.26)	82%
CSF	1	74	1.58 (0.78 to 2.38)	NA
Type of biochemical assay				
SIMOA	1	8	−1.04 (−3.26 to 1.17)	NA
ELISA	5	475	0.84 (0.14 to 1.53)	84%

**Table 3:** Summary of findings

Certainty assessment									Summary of findings		
Participants (studies) Follow-up	Risk of bias	Inconsistency		Indirectness	Imprecision		Publication bias	Overall certainty of evidence	Study event rates		Anticipated absolute effects
									Favorable/Survivors	Unfavorable /non-survivors	Risk difference
**Unfavorable neurological outcome**											
1,648 (10 observational studies)	Serious^a^	Not serious	Not serious		Not serious	Not observed		⨁◯◯◯ Very low	847	801	SMD 0.45 **higher** (0.33 higher to 0.56 higher)
**Mortality**											
483 (6 observational studies)	Serious^a^	Serious^b^	Not serious		Serious^c^	Not observed		⨁◯◯◯ Very low	411	72	SMD 0.71 **higher** (0.04 higher to 1.39 higher)

**CI**: confidence interval; **SMD**: standardized mean difference

^a^ Downgraded for risk of bias due to unclear methodology for patient recruitment and outcome evaluation

^b^ Downgraded for inconsistency because of large heterogeneity (I² = 82%) not explained by subgroup analyses

^c^ Downgraded because confidence intervals were wide, limiting result interpretability

### Secondary outcome

#### Mortality

Six studies (n = 483 participants) [30, 31, 33, 35, 36, 41] were included in the meta-analysis for mortality. There was a significant association between elevated NfL levels and mortality (SMD 0.71 [95% CI 0.04 to 1.39]; I² = 82%) (Figure S4). Subgroup analyses suggested that the association between NfL levels and mortality was more consistent when NfL was measured within the first 24 h after injury (SMD = 0.58[0.18 to 0.98]) with no observed heterogeneity (I² = 0%) (Table 2, Figure S5A). There were no differences in other subgroups analyzed (Table 2, Figure S5B to D). One study not included in the meta-analysis narratively reported a significant predictive value of NfL for mortality [32]. The certainty of evidence for mortality was graded as very low because of serious risk of bias, substantial inconsistency across studies,and imprecision. Inconsistency was driven by the high between-study heterogeneity (I² = 82%), which was not explained by subgroup analyses. Imprecision reflected the wide confidence interval around the pooled estimate, which limited the certainty of the observed association (Table 3).

Other secondary outcomes (length of stay in hospital and intensive care unit, and quality of life) were not reported in any studies.

## Discussion

In our systematic review and meta-analysis, we observed that patients with unfavorable neurological outcome had higher NfL levels compared to those with favorable outcomes; a similar association was observed with mortality, though this finding was accompanied by high heterogeneity across studies and should be interpreted with caution*.*

Our study addresses a distinct research question from previous systematic reviews and meta-analyses on NfL in TBI patients. While earlier reviews showed higher NfL levels in TBI patients compared to healthy or non-TBI controls, they did not investigate its prognostic value within the TBI population [14, 43]. Furthermore, most of these studies analyzed were conducted in patients with mild TBI for diagnostic purpose. In contrast, our work specifically investigates the association between NfL concentrations and outcomes in patients with moderate to severe TBI, a population for whom reliable prognostic tools are needed to help guide clinical decision-making. Our findings are consistent with a growing body of evidence supporting the prognostic role of NfL in various neurological disorders. In ischemic stroke, a recent meta-analysis demonstrated that higher NfL levels are associated with unfavorable long-term functional outcomes [44]. Similar associations have been observed in patients with cardiac arrest, where elevated NfL concentrations correlated with both the severity of anoxic brain injury and neurological recovery [42, 45]. In neurodegenerative diseases, meta-analyses have primarily focused on the diagnostic utility of NfL [8] rather than its prognostic potential. Nevertheless, recent longitudinal cohort studies have reported findings consistent with ours, showing that blood NfL levels are associated with prognosis in Parkinson’s disease [46], amyotrophic lateral sclerosis [47], and multiple sclerosis [48]. Our results are also consistent with systematic reviews and meta-analyses assessing the prognostic value of other cerebral biomarkers in moderate to severe TBI. High serum levels of S100B [5] and NSE [4] have previously been associated with unfavorable neurological outcomes. Specific astrocytic GFAP levels have also been associated with unfavorable neurological outcomes and mortality, consistent with our findings [6]. Nevertheless, as with other brain biomarkers such as GFAP, S100B, and UCH-L1, NfL should not be used as a standalone prognostic biomarker after TBI. Its future clinical value will more likely depend on whether it provides added value beyond other prognostic indicators within multimodal prognostic models.

Timing of NfL sampling is another important factor that may influence its prognostic value. Unlike other biomarkers such as S100B or NSE, NfL displays a slower kinetic profile, with serum levels continuing to rise over the first days or even weeks following injury.[12] This prolonged elevation likely reflects ongoing axonal damage rather than acute secondary insults. In our subgroup analysis, studies with sampling within the first 24 h post-injury showed a stronger association between NfL levels and mortality, suggesting that early and substantial release may indicate severe initial axonal injury and a higher risk of early death. In contrast, no consistent pattern was observed for long-term functional outcomes, possibly due to the slower kinetics of NfL and the heterogeneity in sampling times. A single early measurement may capture the immediate severity of injury associated with death, whereas serial assessments over subsequent days or weeks could better reflect the evolving axonal disruption that influences long-term recovery. Consistent with this, studies incorporating repeated NfL measurements suggested that serum NfL continues to rise over the first days to weeks after injury, with higher trajectories observed in patients with unfavorable outcomes and delayed peaks reported in longitudinal cohorts [30, 32, 40], highlighting that a single measurement may be insufficient to capture the full prognostic information carried by this biomarker. These observations are supported by a recent observational study showing that in patients with moderate to severe TBI, persistently elevated NfL levels are associated with worse neurological outcome [39]. Together, these findings suggest that timing of NfL sampling is a critical determinant of its prognostic value, that universal prognostic thresholds cannot currently be derived across heterogeneous sampling windows, and that future prognostic studies should prioritize standardized sampling schedules and longitudinal analyses. Beyond its association with clinical outcomes, several studies included in this review also support the biological validity of NfL as a marker of structural brain injury [30–32, 36]. Acute serum NfL has been associated with MR-DTI markers of diffuse axonal injury, correlated with CT-based measures of injury severity, and reported at higher levels in patients with CT-visible lesions. These observations are consistent with the known biological role of NfL as an axonal structural protein and support the interpretation that elevated circulating levels primarily reflect the burden of traumatic axonal damage. Although such findings do not fully exclude the influence of other determinants of outcome, they strengthen the plausibility that NfL captures genuine brain structural injury rather than only non-specific critical illness severity.

Our study has several strengths. We developed our protocol using high methodological standards in line with current guidelines for systematic reviews and meta-analyses. Our comprehensive and sensitive search strategy, tailored for prognostic studies and free of language or publication date restrictions, identified all relevant studies across multiple databases. Our conclusions are based on the best currently available evidence and rely on data from 10 studies encompassing a total of 1,648 patients for the primary outcome.

Our study has limitations. One major limitation of cerebral biomarkers is that their levels can be elevated in the presence of extracranial injuries, which may potentially confound their prognostic value. Although NfL is considered highly specific to neuronal damage and minimally influenced by non-cerebral trauma, [7, 11] none of the studies included in our review clearly distinguished between isolated TBI and multiple trauma, limiting our ability to assess the specific impact of extracranial injuries on NfL prognostic performance. Nevertheless, this potential confounding bears more weight when looking at a biomarker for diagnostic purposes in milder TBI compared to more severe TBI for prognostication. The strength of our conclusions is also limited by the quality of the included studies. Many studies exhibited a risk of bias, notably due to the lack of information on patient enrollment methods. Another limitation is assay heterogeneity. Included studies used both ELISA- and SIMOA-based methods, which differ in analytical sensitivity, lower limits of detection, and measurement variability. As a result, absolute NfL concentrations should not be directly compared across studies, and the scarcity of data on assay-specific optimal thresholds for predicting unfavorable neurological outcomes or mortality further limits the clinical interpretability of absolute NfL concentrations across studies. Although the use of SMDs partially mitigates this issue by standardizing within-study effect sizes, residual between-study heterogeneity attributable to assay differences cannot be fully excluded, and pooled estimates should be interpreted with caution*.* Moreover, substantial statistical heterogeneity was observed for mortality outcomes, which was not fully explained by the timing of biomarker sampling. The residual variability may reflect underlying differences in TBI mechanisms (e.g., diffuse axonal injury vs. focal hematoma), patient characteristics or the presence of extracranial injuries. Moreover, mortality outcomes may also have been influenced by variations in decisions regarding withdrawal of life-sustaining therapies, which were not reported and could not be accounted for. Finally, the very low certainty of evidence reflects methodological limitations of current studies rather than an absence of association. Taken together, these limitations indicate that the evidence remains methodologically heterogeneous and insufficiently standardized. Future studies should address these gaps by prospectively enrolling consecutive patients, standardizing the timing and method of NfL measurement, adjusting for confounders, and reporting outcomes according to established prognostic research guidelines*.*

## Conclusions

In our systematic review and meta-analysis, higher NfL concentrations were associated with unfavorable functional outcomes. The association with mortality was more uncertain and should be interpreted with caution given the high heterogeneity across studies and the limited number of non-survivors included. Whether NfL provides additional prognostic information in addition to current prognostic indicators remains to be determined*.*

## Supplementary Material

Supplementary Material 1.

## Data Availability

All data relevant to the study are included in this article or uploaded as supplementary information.

## Abbreviations

CI: Confidence interval
CENTRAL: Cochrane central register of controlled trials
CSF: Cerebrospinal fluid
ELISA: Enzyme-linked immunosorbent assay
GCS: Glasgow coma scale
GFAP: Glial fibrillary acidic protein
GOS: Glasgow outcome scale
GOS-E: Glasgow Outcome scale–extended
GRADE: Grading of Recommendations Assessment, Development and Evaluation
ICU: Intensive care unit
IMPACT: International Mission for Prognosis and Analysis of Clinical Trials in TBI
MRI: Magnetic resonance imaging
NfL: Neurofilament light chain
NSE: Neuron-specific enolase
PRESS: Peer review of electronic search strategies
PRISMA: Preferred reporting items for systematic reviews and meta-analyses
QUADAS-2: Quality assessment of diagnostic accuracy studies 2
RCT: Randomized controlled trial
RevMan: Review manager
SIMOA: Single molecule array
SMD: Standardized mean difference
S100B: S100 calcium-binding protein B
STROBE: Strengthening the reporting of observational studies in epidemiology
TBI: Traumatic brain injury
WLST: Withdrawal of life-sustaining therapies
