# Correction: Association between neurofilament light chain concentrations and outcomes in patients with moderate to severe traumatic brain injury: a systematic review and meta-analysis

**DOI:** 10.1186/s13054-026-06117-3

**Published:** 2026-06-03

**Authors:** Marwan Bouras, Mathieu Pageau, Marc-Aurèle Gagnon, Olivier Costerousse, Karolanne Demers, Anouk Grenier-Gagnon, Chartelin Jean Isaac, Tomas Hayg Torkomyan, François Lauzier, Ryan Zarychanski, Charles L. Francoeur, Peter Gerges, Godwill Abiala, Lynne Moore, Shane W. English, Alexis F. Turgeon

**Affiliations:** 1 CHU de Québec, Population Health and Optimal Health Practices Research Unit Trauma- Emergency-Critical Care Medicine, Université Laval Research Center, Université Laval, Québec City, QC Canada; 2 Department of Anesthesiology and Critical Care Medicine, Division of Critical Care Medicine, Université Laval, Québec City, QC Canada; 3 Department of Anesthesiology and Critical Care Medicine, Centre Hospitalier Régional Universitaire de Brest, Brest, France; 4 Department of Medicine, Université Laval, Québec City, QC Canada; 5 Department of Internal Medicine, Sections of Critical Care Medicine, of Hematology and of Medical Oncology, Rady Faculty of Medicine, University of Manitoba, Winnipeg, MB Canada; 6 Research Institute of Oncology and Hematology, Cancer Care Manitoba, Winnipeg, MB Canada; 7 Department of Preventive and Social Medicine, Université Laval, Québec City, QC Canada; 8 Acute Care Research, Ottawa Hospital Research Institute, Ottawa, ON Canada; 9 Division of Critical Care, Department of Medicine, University of Ottawa, Ottawa, ON Canada

**Correction: Critical Care (2026) 30:234**

10.1186/s13054-026-06036-3

Following publication of the original article [1], the authors identified errors in the author names of Karolanne Demers and Shane W. English.

The incorrect author name is: Karolane Demers.

The correct author name is: Karolanne Demers.

The incorrect author name is: Shane W. Englis.

The correct author name is: Shane W. English.

The author group has been updated above and the original article [1] has been corrected.
