# Supplementary material for: Association between neurofilament light chain concentrations and outcomes in patients with moderate to severe traumatic brain injury: a systematic review and meta-analysis
Source: Crit Care. 2026 May 4;30:234. doi: 10.1186/s13054-026-06036-3 (PMC13147746; doi:10.1186/s13054-026-06036-3)
Supplement: Supplementary file 1 — Supplementary Material 1. [file 13054_2026_6036_MOESM1_ESM.docx]

SUPPLEMENTAL MATERIAL

[Appendix 1. Search strategy 2](#_Toc217198808)

[Figure S1. Risk of bias presented by individual studies (A) and summarized by domain as percentages across included trials (B). 4](#_Toc217198809)

[Figure S2. Funnel Plot for unfavorable neurological outcome 6](#_Toc217198810)

[Figure S3. Subgroup analysis for unfavorable neurological outcome 7](#_Toc217198811)

[Figure S4. Meta-analysis of the association between NfL levels and mortality 9](#_Toc217198812)

[Figure S5. Subgroup analysis for mortality 10](#_Toc217198813)

[Table S1. STROBE reporting guideline checklist 12](#_Toc217198814)

# Appendix 1. Search strategy

MEDLINE (via Ovid)

**1. TBI**

brain injury.TI,AB OR brain injuries.TI,AB OR brain injured.TI,AB OR brain trauma.TI,AB OR brain traumas.TI,AB OR brain traumatic*.TI,AB OR brain traumato*.TI,AB OR brain damag*.TI,AB OR

head injury.TI,AB OR head injuries.TI,AB OR head injured.TI,AB OR head trauma.TI,AB OR head traumas.TI,AB OR head traumatic*.TI,AB OR head traumato*.TI,AB OR head damag*.TI,AB OR

brainstem* injury.TI,AB OR brainstem* injuries.TI,AB OR brainstem* injured.TI,AB OR brainstem* trauma.TI,AB OR brainstem* traumas.TI,AB OR brainstem* traumatic*.TI,AB OR brainstem* traumato*.TI,AB OR brainstem* damag*.TI,AB OR

craniocerebral* injury.TI,AB OR craniocerebral* injuries.TI,AB OR craniocerebral* injured.TI,AB OR craniocerebral* trauma.TI,AB OR craniocerebral* traumas.TI,AB OR craniocerebral* traumatic*.TI,AB OR craniocerebral* traumato*.TI,AB OR craniocerebral* damag*.TI,AB OR

intracrani* injury.TI,AB OR intracrani* injuries.TI,AB OR intracrani* injured.TI,AB OR intracrani* trauma.TI,AB OR intracrani* traumas.TI,AB OR intracrani* traumatic*.TI,AB OR intracrani* traumato*.TI,AB OR intracrani* damag*.TI,AB OR intra-crani* injury.TI,AB OR intra-crani* injuries.TI,AB OR intra-crani* injured.TI,AB OR intra-crani* trauma.TI,AB OR intra-crani* traumas.TI,AB OR intra-crani* traumatic*.TI,AB OR intra-crani* traumato*.TI,AB OR intra-crani* damag*.TI,AB OR

intercrani* injury.TI,AB OR intercrani* injuries.TI,AB OR intercrani* injured.TI,AB OR intercrani* trauma.TI,AB OR intercrani* traumas.TI,AB OR intercrani* traumatic*.TI,AB OR intercrani* traumato*.TI,AB OR intercrani* damag*.TI,AB OR inter-crani* injury.TI,AB OR inter-crani* injuries.TI,AB OR inter-crani* injured.TI,AB OR inter-crani* trauma.TI,AB OR inter-crani* traumas.TI,AB OR inter-crani* traumatic*.TI,AB OR inter-crani* traumato*.TI,AB OR inter-crani* damag*.TI,AB OR cerebral* injury.TI,AB OR cerebral* injuries.TI,AB OR cerebral* injured.TI,AB OR cerebral* trauma.TI,AB OR cerebral* traumas.TI,AB OR cerebral* traumatic*.TI,AB OR cerebral* traumato*.TI,AB OR cerebral* damag*.TI,AB OR cerebel* injury.TI,AB OR cerebel* injuries.TI,AB OR cerebel* injured.TI,AB OR cerebel* trauma.TI,AB OR cerebel* traumas.TI,AB OR cerebel* traumatic*.TI,AB OR cerebel* traumato*.TI,AB OR cerebel* damag*.TI,AB OR

forebrain* injury.TI,AB OR forebrain* injuries.TI,AB OR forebrain* injured.TI,AB OR forebrain* trauma.TI,AB OR forebrain* traumas.TI,AB OR forebrain* traumatic*.TI,AB OR forebrain* traumato*.TI,AB OR forebrain* damag*.TI,AB OR TBI.TI,AB OR Craniocerebral Trauma.sh OR Brain Injuries.sh OR Brain Hemorrhage, Traumatic.sh OR Diffuse Axonal Injury.sh OR Coma, Post-Head Injury.sh OR Head Injuries, Closed.sh OR Intracranial Hemorrhage, Traumatic.sh

**2. Biomarker**

NFL.ti,ab OR

NF-L.ti,ab OR

NEFL.ti,ab OR

Neurofilament light.ti,ab OR

Neurofilament L.ti,ab OR

Light neurofilament.ti,ab OR

Neurofilament protein light.ti,ab OR

Neurofilament protein L.ti,ab OR

Neurofilament triplet L.ti,ab OR

Neurofilament 68.ti,ab OR

NF68.ti,ab OR

NF-68.ti,ab OR

68kDa neurofilament.ti,ab OR

68 kDa neurofilament.ti,ab

**3.** 1 AND 2

**4**. animal*.sh NOT human*.sh

**5**. 3 NOT 4

# **Figure S1.** Risk of bias presented by individual studies (A) and summarized by domain as percentages across included trials (B).

A.

**
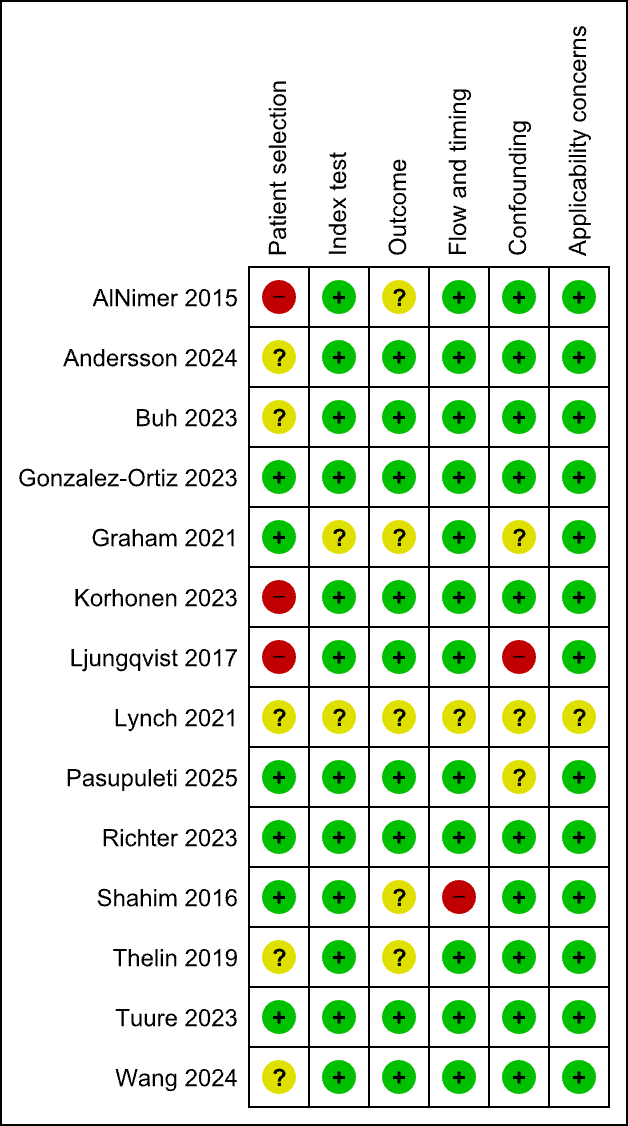
**

B.

**
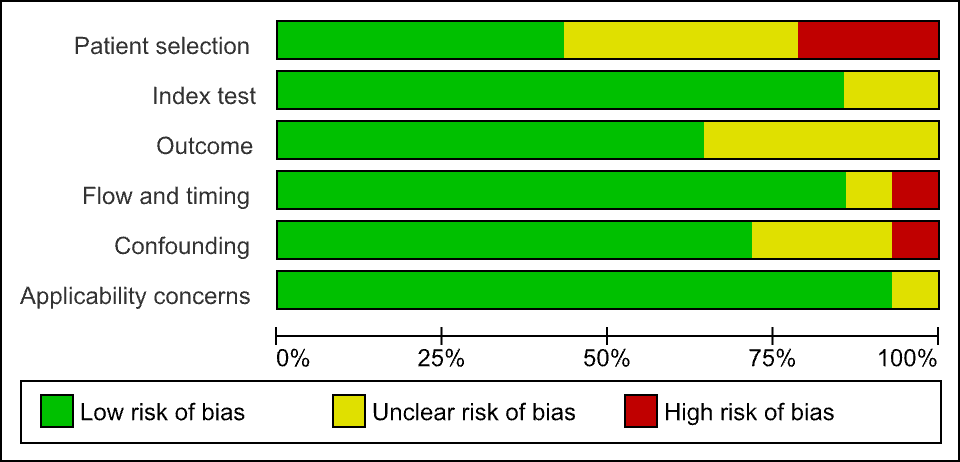
**

# **Figure S2.** Funnel Plot for unfavorable neurological outcome

SMD: Standard Mean Difference

# **Figure S3.** Subgroup analyses for unfavorable neurological outcome

1. Sampling time
2. TBI severity
3. Type of sample

1. Type of assay

# **Figure S4.** Association between NfL levels and mortality

**
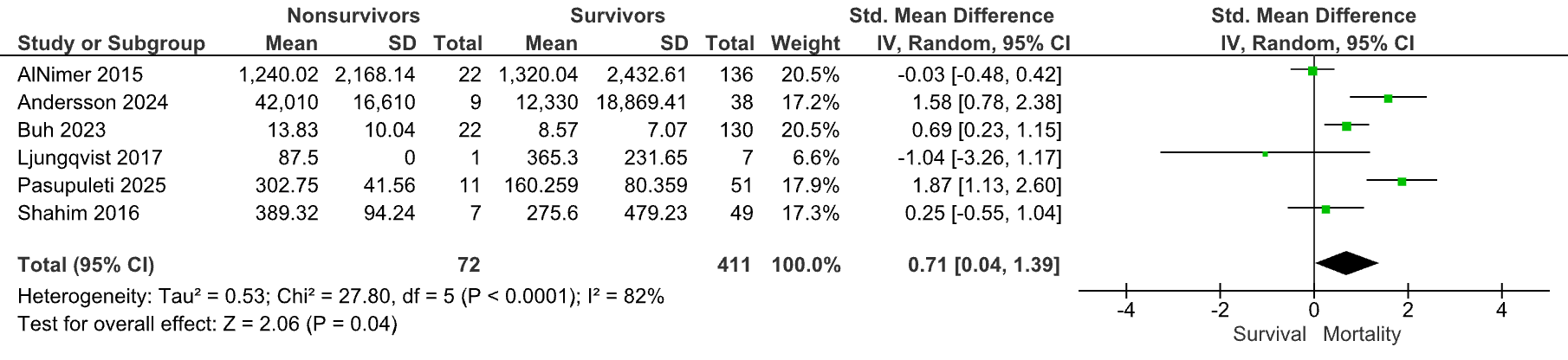
**

CI = confidence interval

SD = standard deviation

IV = inverted variance

# **Figure S5.** Subgroup analyses for mortality

1. Sampling time


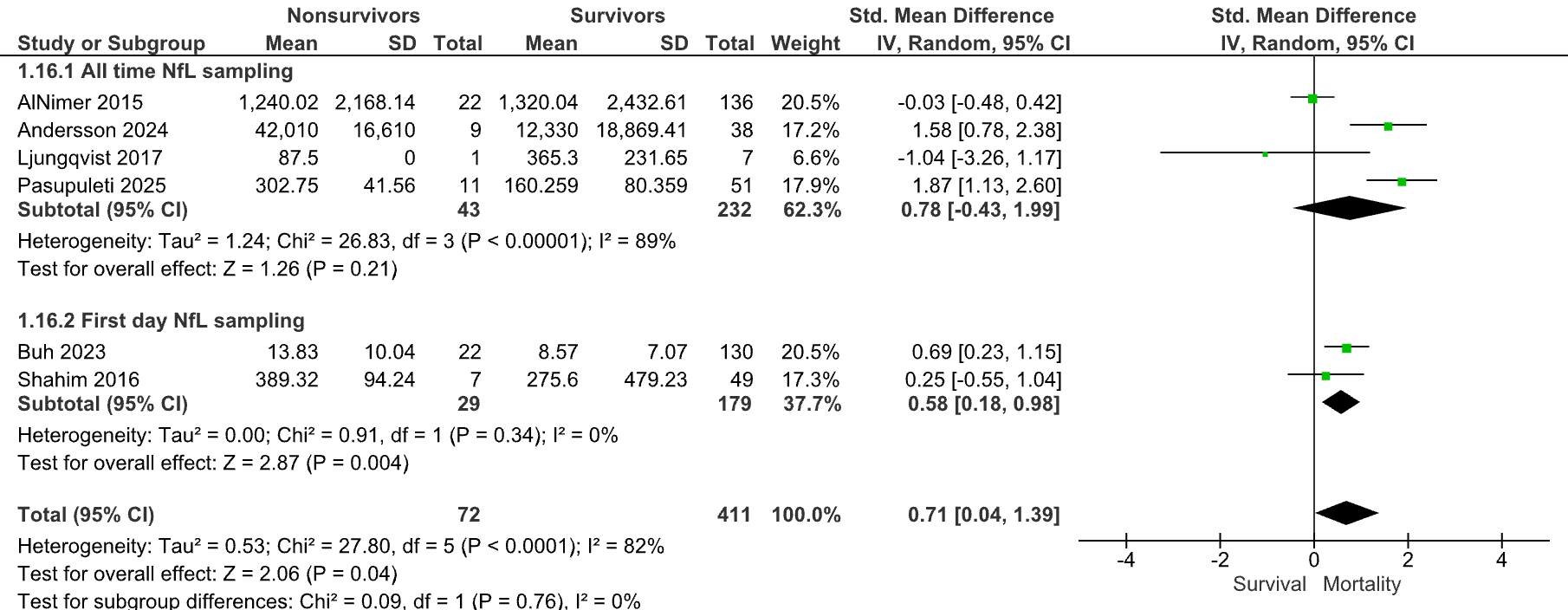


1. TBI severity


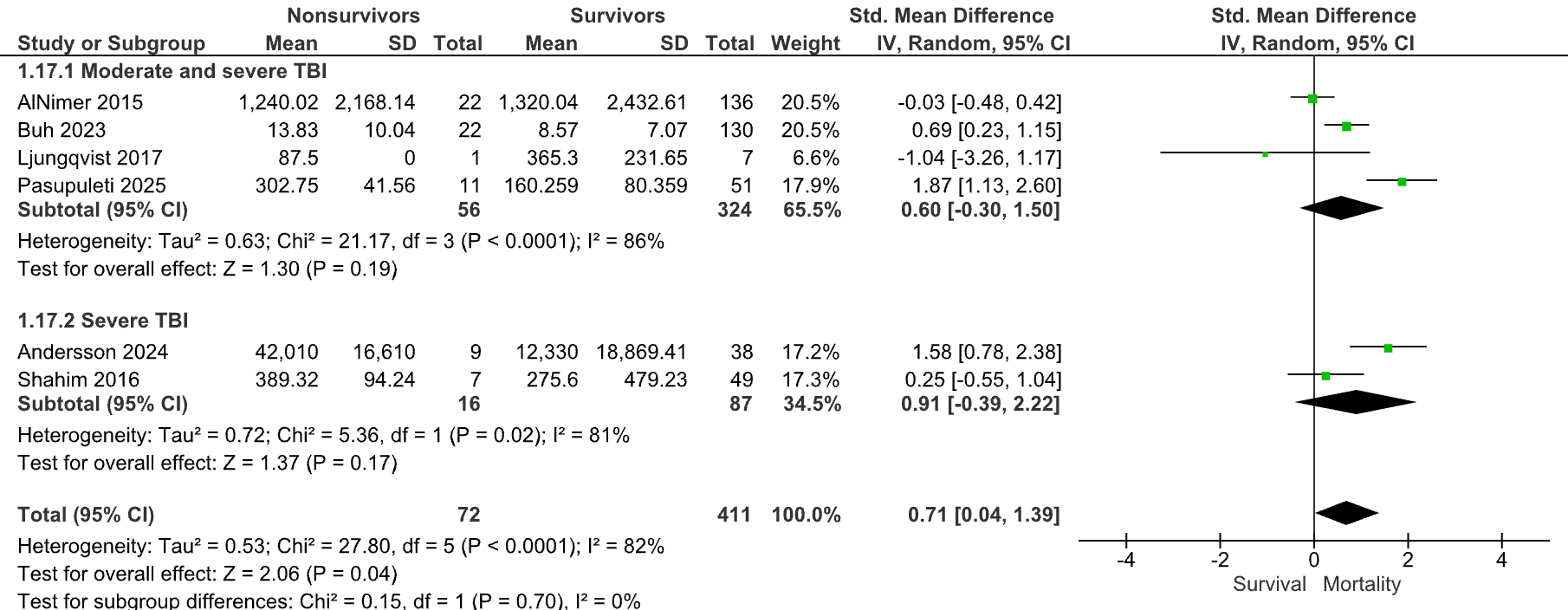


1. Type of sample


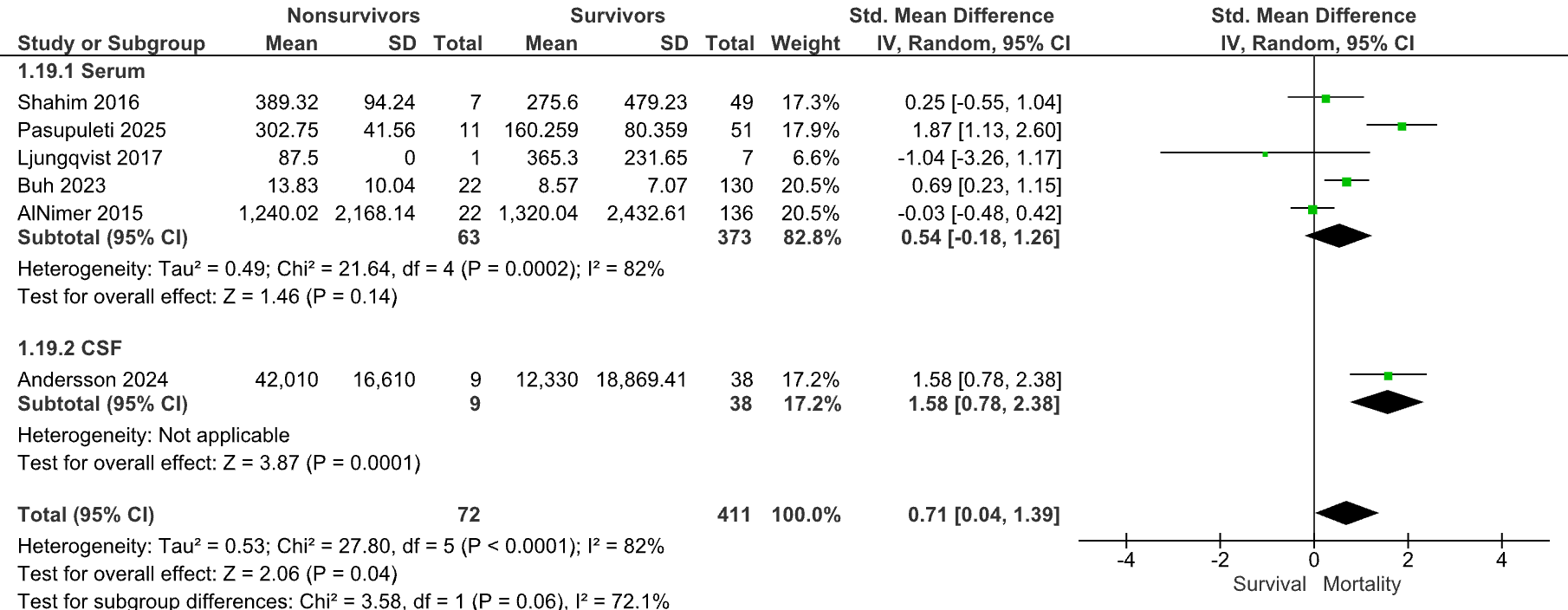


1. Type of assay


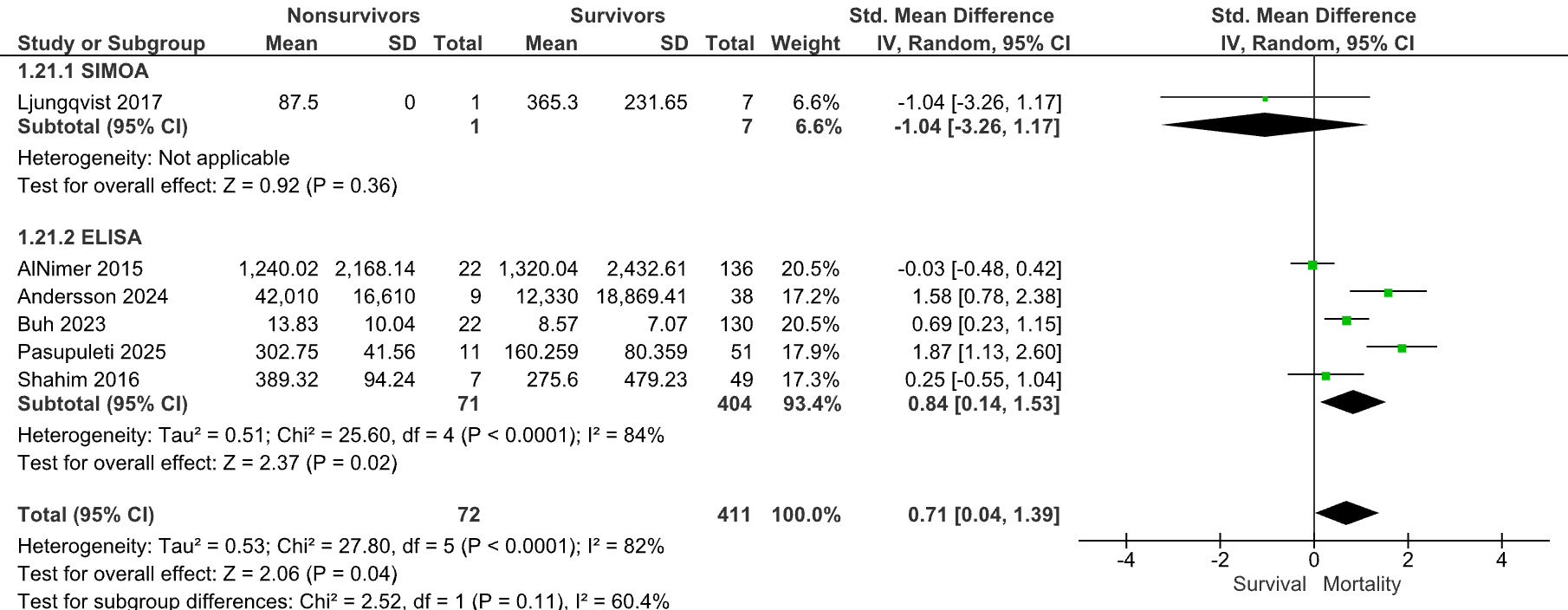


# **Table S1.** STROBE reporting guideline checklist

|  | **Ljungqvist 2017** | **Thelin 2019** | **AlNimer 2015** | **Shahim 2016** | **Graham 2021** | **Lynch 2021** | **Gonzalez-Ortiz 2023** | **Korhonen**  **2023** | **Richter**  **2023** | **Tuure**  **2023** | **Wang**  **2023** | **Robba 2024** | **Anderson 2024** | **Buh 2023** | **Pasupuleti**  **2025** |
| --- | --- | --- | --- | --- | --- | --- | --- | --- | --- | --- | --- | --- | --- | --- | --- |
| **Title and abstract** |  |  |  |  |  |  |  |  |  |  |  |  |  |  |  |
| **Study Design** | Yes | No | No | Yes | No | Yes | Yes | Yes | Yes | Yes | No | No | No | No | No |
| **Abstract** | Yes | Yes | Yes | Yes | Yes | Yes | Yes | Yes | Yes | Yes | Yes | Yes | Yes | Yes | Yes |
| **Introduction** |  |  |  |  |  |  |  |  |  |  |  |  |  |  |  |
| **Background** | Yes | Yes | Yes | Yes | Yes | No | Yes | Yes | Yes | Yes | Yes | Yes | Yes | Yes | Yes |
| **Objectives** | Yes | Yes | Yes | Yes | Yes | No | Yes | Yes | Yes | Yes | Yes | Yes | Yes | Yes | Yes |
| **Methods** |  |  |  |  |  |  |  |  |  |  |  |  |  |  |  |
| **Study design** | Yes | Yes | Yes | Yes | Yes | Yes | Yes | Yes | Yes | Yes | Yes | Yes | Yes | Yes | Yes |
| **Setting** | Yes | Yes | Yes | No | Yes | No | Yes | Yes | Yes | Yes | Yes | Yes | Yes | Yes | Yes |
| **Participants** | Yes | No | Yes | Yes | Yes | No | Yes | Yes | Yes | Yes | Yes | Yes | Yes | Yes | Yes |
| **Variables** | Yes | Yes | Yes | Yes | Yes | No | Yes | Yes | Yes | Yes | Yes | Yes | Yes | Yes | Yes |
| **Data sources/measurement** | Yes | Yes | Yes | Yes | Yes | No | Yes | Yes | Yes | Yes | Yes | Yes | Yes | Yes | No |
| **Bias** | No | Yes | Yes | No | No | No | No | No | Yes | Yes | Yes | Yes | Yes | Yes | Yes |
| **Study size** | No | No | No | No | Yes | No | No | No | No | No | Yes | No | No | No | No |
| **Quantitative variables** | Yes | Yes | Yes | No | Yes | No | Yes | Yes | Yes | Yes | Yes | Yes | Yes | Yes | Yes |
| **Statistical methods** |  |  |  |  |  |  |  |  |  |  |  |  |  |  |  |
| **Control for cofounding** | Yes | Yes | Yes | No | Yes | No | Yes | No | No | Yes | Yes | Yes | Yes | No | Yes |
| **Subgroups and interaction** | No | No | Yes | No | No | No | No | No | No | Yes | No | No | No | No | No |
| **Missing data** | No | Yes | Yes | Yes | No | No | NA | Yes | Yes | No | No | No | No | No | No |
| **Lost to follow-up** | No | No | Yes | Yes | No | No | NA | NA | NA | No | No | No | No | No | NA |
| **Sensitivity analyses** | No | No | No | No | Yes | No | NA | No | No | No | No | No | No | No | No |
| **Results** |  |  |  |  |  |  |  |  |  |  |  |  |  |  |  |
| **Participants** |  |  |  |  |  |  |  |  |  |  |  |  |  |  |  |
| **Numbers at each stage** | No | No | No | No | Yes | No | NA | No | NA | NA | No | Yes | No | No | Yes |
| **Lost at each stage** | Yes | No | No | Yes | Yes | No | NA | NA | No | No | No | Yes | No | No | NA |
| **Flow diagram** | No | No | No | Yes | Yes | No | No | No | No | No | No | No | No | No | No |
| **Descriptive data** |  |  |  |  |  |  |  |  |  |  |  |  |  |  |  |
| **Characteristics of participants** | Yes | Yes | Yes | Yes | Yes | No | Yes | Yes | Yes | Yes | Yes | Yes | Yes | Yes | Yes |
| **Missing data** | Yes | Yes | Yes | Yes | No | No | NA | NA | No | No | No | No | No | No | No |
| **Follow-up time** | Yes | Yes | Yes | Yes | Yes | No | Yes | NA | NA | NA | Yes | Yes | Yes | Yes | Yes |
| **Outcome data** | Yes | Yes | Yes | Yes | Yes | No | Yes | Yes | Yes | Yes | Yes | Yes | Yes | Yes | Yes |
| **Main results** |  |  |  |  |  |  |  |  |  |  |  |  |  |  |  |
| **Estimates and precision** | Yes | Yes | Yes | Yes | Yes | No | Yes | NA | Yes | Yes | Yes | Yes | Yes | Yes | Yes |
| **Category boundaries** | Yes | Yes | Yes | Yes | No | No | NA | NA | NA | NA | Yes | Yes | Yes | Yes | Yes |
| **Translating RR in absolute risk** | NA | NA | NA | NA | NA | NA | No | NA | NA | NA | No | No | No | No | NA |
| **Other analyses** | No | Yes | Yes | No | Yes | No | No | No | Yes | Yes | Yes | Yes | Yes | No | No |
| **Discussion** |  |  |  |  |  |  |  |  |  |  |  |  |  |  |  |
| **Key results** | Yes | Yes | Yes | Yes | Yes | Yes | Yes | Yes | Yes | Yes | Yes | Yes | Yes | No | No |
| **Limitations** | Yes | Yes | Yes | Yes | Yes | No | Yes | Yes | Yes | Yes | Yes | Yes | Yes | Yes | Yes |
| **Interpretation** | Yes | Yes | Yes | Yes | Yes | Yes | Yes | Yes | Yes | Yes | Yes | Yes | Yes | Yes | Yes |
| **Generalizability** | Yes | Yes | Yes | Yes | No | No | Yes | Yes | Yes | Yes | Yes | Yes | No | Yes | No |
| **Other** |  |  |  |  |  |  |  |  |  |  |  |  |  |  |  |
| **Fundings** | Yes | Yes | Yes | Yes | Yes | No | Yes | Yes | Yes | Yes | Yes | Yes | Yes | Yes | Yes |
| **Total items missing** | N = 9 (28%) | N = 9 (28%) | N = 6 (19%) | N = 10 (31%) | N = 9 (28%) | N = 28 (85%) | N =12 (36%) | N=14  (42%) | N =12 (36%) | N =11 (33%) | N =10 (30%) | N =9 (27%) | N =12  (36%) | N =14  (42%) | N =11  (33%) |
